# The Role of Defensins in HIV Pathogenesis

**DOI:** 10.1155/2017/5186904

**Published:** 2017-08-03

**Authors:** Barcley T. Pace, Andrew A. Lackner, Edith Porter, Bapi Pahar

**Affiliations:** ^1^Division of Comparative Pathology, Tulane National Primate Research Center, Covington, LA, USA; ^2^Tulane University School of Medicine, New Orleans, LA, USA; ^3^Department of Biological Sciences, California State University, Los Angeles, Los Angeles, CA, USA

## Abstract

Profound loss of CD4^+^ T cells, progressive impairment of the immune system, inflammation, and sustained immune activation are the characteristics of human immunodeficiency virus-1 (HIV-1) infection. Innate immune responses respond immediately from the day of HIV infection, and a thorough understanding of the interaction between several innate immune cells and HIV-1 is essential to determine to what extent those cells play a crucial role in controlling HIV-1 *in vivo*. Defensins, divided into the three subfamilies *α*-, *β*-, and *θ*-defensins based on structure and disulfide linkages, comprise a critical component of the innate immune response and exhibit anti-HIV-1 activities and immunomodulatory capabilities. In humans, only *α*- and *β*-defensins are expressed in various tissues and have broad impacts on HIV-1 transmission, replication, and disease progression. *θ*-defensins have been identified as functional peptides in Old World monkeys, but not in humans. Instead, *θ*-defensins exist only as pseudogenes in humans, chimpanzees, and gorillas. The use of the synthetic *θ*-defensin peptide “retrocyclin” as an antiviral therapy was shown to be promising, and further research into the development of defensin-based HIV-1 therapeutics is needed. This review focuses on the role of defensins in HIV-1 pathogenesis and highlights future research efforts that warrant investigation.

## 1. Introduction

Since the start of the epidemic in the 1980s, 35 million people have died and more than 70 million have been infected from human immunodeficiency virus-1 (HIV-1) infection [1]. The primary manner of HIV-1 transmission occurs at mucosal surfaces [2, 3], including the oral [4], cervicovaginal [5], and rectal mucosal epithelia [6]. HIV-1 predominantly targets cells associated with the adaptive immune response, in particular CD4^+^ T cells [7], which reside primarily in the lymph nodes and gastrointestinal tract [8, 9]. Immune activation induced by HIV-1 infection provides more CD4^+^ T cell targets for viral replication, increases T cell turnover and depletion, and eventually initiates a vicious cycle of uncontrolled viral replication [8]. At this stage of infection, the failing immune system allows for the reemergence of preexisting, latent pathogens that further burden immune responses [10]. The formation of this vicious cycle leads to exhaustion of the adaptive immune system and eventual progression to acquired immune deficiency syndrome (AIDS). Although the adaptive immune response plays a large role in HIV-1 pathogenesis and progression to AIDS, mounting evidence suggests that the innate immune system directly or indirectly impacts disease progression [11]. Myeloid cells of the innate immune system including monocytes, macrophages, and dendritic cells (DCs) are also targeted by the virus [12]. In early stages of HIV-1 infection, evidence suggests that DCs initially transmit HIV-1 across mucosal barriers [3]. The exact time course of disease progression is difficult to predict, owing to variation in factors such as host genetics and the environment. Two to four weeks may pass following initial exposure to HIV-1 before full activation of the adaptive immune response is initiated [13]. During this time, the virus replicates and spreads without much control via infected CD4^+^ T cells [8]. This unregulated viral replication suggests a failure of innate immune mechanisms, such as natural killer cells that normally control viral infections, whose cytolytic function is compromised during HIV-1 viremia [14]. In rhesus macaques, rapid upregulation of inflammasome following simian immunodeficiency virus (SIV) infection was shown to be responsible for the dysregulation of innate and acquired immune responses [11, 15]. When finally activated, CD8^+^ T cells dramatically decrease HIV-1 viremia. However, at this stage of infection, the virus has already established a reservoir for persistent, low-level replication in mucosa-associated lymphatic tissue, in particular in the gut, and the host becomes chronically infected [8]. It is unclear whether innate immunity contributes to the reduced viral replication and regulates immune activation.

One critical component of the innate response includes a family of small, antimicrobial peptides termed defensins [16]. Defensins are cationic peptides characterized by a *β*-sheet structure and three intramolecular cysteine-disulfide bonds [17]. Vertebrate defensins are comprised of *α*, *β*, and *θ* subfamilies, with each differing in both the size and pattern of disulfide linkages [18]. *α*-Defensins can be further subdivided into two classes, myeloid and enteric *α*-defensins. Myeloid *α*-defensins, consisting of human neutrophil peptides (HNP) 1–4, reside within primary granules of neutrophils and are synthesized in the bone marrow (Table 1) [16, 19, 20]. There have been conflicting reports on the presence of HNPs in T lymphocytes and other cell types, but while, under certain circumstances, genuine expression may occur, the presence seems to be rather a consequence of pinocytic uptake [21]. The enteric *α*-defensins, human defensins (HD) 5 and 6, are both produced by Paneth cells (PCs) in the crypts of the small intestine, while HD5 is also synthesized by epithelial cells of the genitourinary tract [22–24]. Human *β*-defensins (HBDs) are expressed by skin and mucosal epithelial cells lining organs such as those in the urinary tract, kidney, and trachea (Table 2) [18, 25]. Numerous HBDs have been described at the genomic level and some at the protein level including HBD1–4 [26–28]. Both *α*- and *β*-defensins can also be expressed in some monocytes, macrophages, and DCs [29]. Unlike *α*- and *β*-defensins, *θ*-defensins possess a cyclical structure and exist only in certain nonhuman primate species, most notably in Old World monkeys and macaques, but not in chimpanzees or gorillas. Rhesus *θ*-defensins (RTDs) exhibiting strong antimicrobial activity, including anti-HIV activity [30], are primarily synthesized in the bone marrow and are expressed by neutrophils, monocytes [29], and PCs (Table 3) [31]. For RTD1, strong anti-inflammatory activity has been described [32]. Although humans (and chimpanzees) possess ancestral genes for *θ*-defensins, these are not expressed due to a stop codon in the preprocoding sequence of *θ*-defensin genes. However, the chemically synthesized ancestral human RTD peptides “retrocyclins” (RCs) have shown great promise for research into antiviral therapies, drug development, and regulatory immune functions [33]. While defensins serve as strong microbicidal agents, they also aid in the regulation of certain facets of the adaptive immune system, including chemotaxis of T cells and monocytes [34–36] and maturation of DCs [37, 38]. On the other hand, some are downregulators of the immune response such as RTD1, which interferes with NF-*κ*B signaling ablating immune activation [32]. This brief review will focus on the role of defensins in HIV-1 pathogenesis and disease progression and as effectors of innate immune responses and modulators of adaptive immune responses.

## 2. *α*-Defensins

### 2.1. HNP1–4

The HNP1–4 defensins affect a wide range of responses to infections with pathogenic microbes. Among these, inhibition of HIV-1 infection was shown to be mediated by blocking viral entry into cells and interfering with critical steps of viral replication following infection *in vitro*. HNP1–3 prevent HIV-1 entry into cells by interfering with the binding of viral gp120 to the CD4^+^ T cells [39–42]. HNP1 can also inhibit HIV-1 replication via the disruption of the protein kinase C signaling pathway in an HIV-infected cell [40]. However, the binding of HNPs to HIV-1 envelope is compromised in the presence of serum due to the high binding affinity of HNPs for serum proteins [43, 44]. This suggests that HNPs may not effectively bind to the virions circulating in the blood. Nonetheless, in breast milk, when adjusted for HIV RNA quantity, HIV-1-positive women with higher concentrations of HNP1–3 were less likely to transmit the virus to their child compared to HIV-1-positive women with lower concentrations [45]. Thus, the anti-HIV activity of HNP1–3 is likely more important in regions with low concentrations of serum proteins, such as at mucosal surfaces [43]. While the ability of HNP1–3 to block viral entry is attenuated in the presence of serum proteins, research demonstrates that HNP4 remains active against HIV-1 by binding gp120 and CD4 regardless of the presence of serum proteins [46]. The ability of HNP4 to limit viral infection may be attributable to the higher binding affinities of HNP1–3 for serum proteins compared to that of HNP4 or unique properties of HNP4 that enable it to interact with different regions of gp120 and CD4 than those that bind HNP1–3. The structure and properties of HNP4 differ dramatically from those of HNP1–3 [47], which may further contribute to the observed differences in HNP activity against HIV-1. While HNP4 remains active against HIV-1 *in vitro*, this effect may be biologically negligible *in vivo* due to the scarce amount of HNP4 produced by neutrophils [47].

Although neutrophils are not prominently represented in HIV-mediated gastroentropathy, HNP1 had been recently shown to reduce tight junction expression in intestinal epithelial cells and promote HIV traversal, adding to the complexity of HNPs' role in the HIV infection process [48].

### 2.2. HD5-6

The direct effects of HD5-6 on HIV-1 transmission in the gastrointestinal (GI) tract and genital mucosa are complex. Because HIV-1 can establish infection within hours after traversing mucosal barriers [3], the ability of innate immune factors to mount a vigorous and immediate response may be the key to successfully preserve intact mucosal barriers and limit viral spread to other tissues. Unlike HNP1, HD5 does not disrupt the intestinal epithelial cell barrier [48], and intestinal PCs constitutively secrete HD5 and 6 to protect the host against invading pathogens and to maintain commensal microbial communities [49–51]. The clinical significance of this role has been well documented for Crohn's disease, which is characterized by Paneth cell dysfunction with reduced HD5 production, alteration of the resident microbiota, subsequent inflammation, and T cell-mediated immune responses [49]. An upregulation of HD5 in the colorectal mucosa has been observed in patients with HIV-1, possibly in response to intestinal inflammation [52]. Similarly, in response to bacterial vaginosis, the synthesis of HD5 (and HBDs) increased, in agreement with the role of defensins in protecting mucosal barriers [53]. HD5 has been demonstrated to have direct inhibitory effects on HIV-1 entry into purified peripheral blood CD4^+^ T lymphocytes by binding with viral gp120 and the CD4 receptor in serum-free condition *in vitro* [54]. However, Ding and colleagues showed that the antiviral effect of HD5 on HIV infection in serum-free primary CD4^+^ T lymphocyte cultures was a result of defensin-mediated cell death and was independent of HIV receptors [55]. Moreover, HD5 treatment enhanced HIV infectivity of HeLa-CD4-CCR5 cells in serum-free condition *in vitro*, in the absence of defensin-mediated cell death [55]. Recent research findings from a mouse enteric adenovirus model revealed that Paneth cell defensins are required for a protective neutralizing antibody response against oral viral infection, suggesting interaction between enteric defensins and the adaptive immune response. Apart from defensin-triggered enhanced immunogenicity of the viral particle through formation of larger aggregates, enteric defensins may chemoattract immune cells and alter T helper and B cell functions. Further studies are needed to determine if enteric defensins similarly recruit T cells in the gut, thereby inducing the gut-associated HIV reservoir.

### 2.3. Effects on Inflammation and HIV-1 Progression

One intriguing area of research that warrants further investigation is the role of *α*-defensins in mitigating the effects of HIV-1 as the disease progresses. HIV-1 and SIV infection in human and animals, respectively, induce inflammation by upregulating proinflammatory cytokine and chemokine expression and increase intestinal permeability in the GI tract [56–58]. Understanding the causes and consequences of continuous immune activation and resulting inflammation associated with HIV-1 pathogenesis has become a focus of current research efforts. These pathologic changes occur in acute or chronic stage infection, and activation of immune responses has been implicated in the development of the enteropathy characteristic of HIV-1 [59]. For example, infection of polarized T84 intestinal epithelial cells with various HIV-1 strains led to an upregulation of TNF*α*, which in turn led to disruption of tight junction proteins and increased permeability, and TNF*α* produced by HIV-1-infected monocyte-derived macrophages impaired barrier function in HT-29/B6 cells by inducing apoptosis [52, 53].

Similarly, the upregulation of IFN*γ* and TNF*α* in SIV-infected macaques promotes apoptosis of intestinal epithelial cells [57]. In humans, the upregulation of intestinal IFN*γ* stimulates PC degranulation, and the subsequent release of HD5 and 6 may promote barrier integrity but nevertheless could increase viral transmission rates due to increased viral uptake [60, 61]. In SIV-infected macaques, enteric *α*-defensin expression was increased in the gut at all stages of infection compared to that in control, suggesting that innate defenses are attempting to compensate for the viral-induced epithelial damage and the effects of mucosal T cell depletion [62]. Once intestinal barriers are compromised, microbes that normally inhabit the gut can cross the epithelium and circulate in the systemic immune system (microbial translocation), which is partly responsible for the persistent immune activation associated with HIV-1/SIV disease progression [63, 64]. The continual immune activation eventually exhausts resources to replenish depleted T cell populations and promotes HIV disease progression. Synthesis of enteric defensins certainly functions to protect the host by preventing intestinal barrier disruption; however, there is a lack of information on the potential immunological impacts of these peptides. Decreases in enteric defensin protein levels were observed in SIV-infected macaques at an advanced stage of infection and correlated with an increase in opportunistic bacterial infections [62]. In SIV-infected sooty mangabeys (SMs), a natural host of SIV, *α*- and *θ*-defensin expression levels were elevated compared to those in SIV-infected macaques, and the lack of disease progression despite high levels of viral replication observed in SMs compared to macaques was in part attributed to an active downregulation of inflammation in SMs [65]. In response to bacterial infection, HNPs reduce proinflammatory cytokine concentration *in vivo* in blood in mice [66]. A recent study demonstrated that HNP1, which retains antimicrobial activity when released from apoptotic neutrophils, inhibits, after uptake, mRNA translation in macrophages and reduces inflammatory exudate formation *in vitro* [66, 67]. However, there is no evidence for elevated gastrointestinal HNP1 concentrations in HIV patients, and a lack of HNP-mediated control of macrophage-driven inflammation may contribute to the development of the continuous inflammation seen in HIV gastroenteropathy.

The antimicrobial activity of *α*-defensins, combined with their effects on immune regulation and response, necessitates further investigation into the complex relationship between these peptides and HIV-1 pathogenesis.

## 3. *β*-Defensins

### 3.1. *In Vitro* Study

*β*-Defensins are predominantly expressed by epithelial cells and therefore can serve as a first line of defense against invading pathogens at mucosal surfaces and skin. The synthesis of HBDs is regulated by, and can regulate, responses of both innate and adaptive immunities. HBD production and secretion are stimulated by microbes [68, 69] and the release of cytokines including IFN*γ*, IL1, IL17A, IL22, and TNF*α* [70–72]. The effects of *β*-defensins on HIV-1 pathogenesis have traditionally centered on the oral environment because the rate of oral HIV-1 transmission is significantly less than that of vaginal or rectal transmission [73, 74], and detectable levels of HBD1, 2, and 3 are routinely found in normal oral epithelium of adults [75]. In addition, recombinant HBD2 and 3 were shown to directly inactivate HIV-1 [76]. A recent study in tonsil epithelial cells has demonstrated that simultaneous binding of heparan sulfate proteoglycans of epithelial cells to HBDs and viral gp120 initiates cointernalization of the defensins and the virions into endosomes and results in reduced HIV infectivity [77]. Following viral entry, HBD2 also blocks HIV-1 replication by preventing an accumulation of reverse transcription products [74]. Moreover, HBD2 and 3 exert anti-HIV-1 activity against both CCR5 and CXCR4 tropic HIV infections [74]. Mother-to-child transmission of HIV-1 most commonly occurs at the oral and gastrointestinal epithelia [78]. In fetal and infant oral epithelial tissues, a lack of HBD expression allows transmigration of virions within oral mucosa and increases the risk of HIV-1 transmission [79]. Both cell-free and cell-associated HIV-1 viral particles can transmigrate through fetal, but not adult, oral epithelium and infect permissive cells [80], further highlighting the potent antiviral activity of HBDs in the adult oral environment.

Coculture experiments with epithelial cells, stromal fibroblasts, and CD4^+^ T cells have shown that epithelial-derived antimicrobial factors can protect CD4^+^ T cells from HIV infection. Endometrial epithelial cells (eEC) significantly overexpressed six genes associated with anti-HIV-1 activity, the most abundant of which were secretory leukocyte peptidase inhibitor and HBD2. The increased gene expression observed in eEC potently inhibited HIV-1 infection of CD4^+^ T cells [81]. However, this study also showed that in the absence of epithelial cells, stromal fibroblasts markedly enhanced HIV infection of CD4^+^ T cells, highlighting the importance of the epithelial cell barrier.

Apart from direct antimicrobial activities, HBD2 has prominent immunomodulatory activities including recruitment of immune cells and induction of antiviral proteins. The first described was the chemotactic activity of HBD2 recruiting not only immature DCs but also memory CD4^+^ T cells by binding to the chemokine receptor CCR6 and later on the chemotaxis induced by HBD2 and HBD3 through CCR2 on myeloid cells [34, 35]. HIV-1 replication is inhibited postentry in PBMCs treated with HBD2 as documented by the inhibition of the accumulation of reverse transcription products [82]. HIV/SIV infection selectively targets and depletes CCR6^+^ CD4^+^ T cells from peripheral blood and those populations that cannot be restored or maintained by antiretroviral treatment [83–86]. However, the interaction between HBD2 and its receptor CCR6 induces the expression of an antiviral protein, the host restriction factor apolipoprotein B mRNA-editing enzyme-catalytic polypeptide-like 3G (APOBEC3G) [82], an enzyme known to prevent complete synthesis of HIV-1 reverse transcripts [87]. CCR6 is expressed on DCs and memory CD4^+^ and CD8^+^ T cells, indicating a potentially important role for HBD2 in preventing HIV-1 infection in CCR6^+^ target cells through the upregulation of additional innate antiviral factors. Other effects include the induction of not only proinflammatory cytokines but also the anti-inflammatory cytokine IL10 and the suppression of IL17 production in CD3/CD28-stimulated T cells [88]. Thus, any alteration in the physiological concentration of HBD2 is likely to have a multitude of effects on the local homeostasis, which is also influenced by the presence of other defensins.

### 3.2. *In Vivo* Studies

*β*-Defensin synthesis has been shown to be associated with the maintenance of GI health following pathogenic infection or the development of intestinal disorders. HBD1 is constitutively expressed in the intestinal epithelium and colon, while HBD2 expression is induced in the colon and duodenum following pathogen exposure [89]. During HIV-1 infection, the extent of microbial translocation that occurs correlates with the amount of inflammation and GI epithelial damage [64]. One proposed reason for the increased intestinal damage in HIV-1-infected patients is the progressive loss of Th17 cells and subsequent diminished control of the resident microbiota by epithelial cells and neutrophils [90]. Th17 cells secrete cytokines that regulate mucosal immune responses and promote secretion of antimicrobial peptides [71], including HBD2 [91]. In SIV infection, a depletion of Th17 cells in the ileal mucosa resulted in increased microbial translocation [92]. The loss of Th17 cells could lead to reduced innate defensin levels triggering additional alteration of immunological signals, thereby exacerbating epithelial damage and translocation of microbes that ultimately induces systemic immune activation in HIV-1-infected individuals. Similar to HIV-1 enteropathy, inflammation induced by irritable bowel disease and ulcerative colitis results in damage to the intestinal epithelium and the subsequent translocation of microbes [93]. HBD2 expression increases in patients with irritable bowel disease [94] and ulcerative colitis [95]. Conversely, HBD1 expression decreases in ulcerative colitis and Crohn's disease [95]. The observed variation in intestinal HBD expression highlights the need for future studies to more thoroughly investigate the underlying mechanisms responsible for regulating antimicrobial synthesis in response to GI disorders and pathogenic infections.

Preexisting genital infections affect inflammatory molecule secretions and HBD synthesis, with downstream effects on HIV-1 disease progression. In women with human papillomavirus (HPV) and HIV-1, HBD2 and proinflammatory cytokine levels were elevated compared to those in HIV-1-positive women without HPV [96]. Similarly, a recent study found that greater *Escherichia coli* inhibitory activity and higher concentrations of HBD1 in cervicovaginal lavage (CVL) were associated with an increased risk of HIV-1 acquisition [97], possibly due to increased mucosal inflammation. An analogous study observed that women who seroconverted to HIV-1 were more likely to have greater *E. coli* bactericidal activity and higher concentrations of HBD2 in vaginal fluid prior to seroconversion compared to women who did not seroconvert [98]. Conversely, women with cervical intraepithelial neoplasia resulting from HPV infection had significantly greater concentrations of proinflammatory cytokines but exhibited lower levels of HBD2 and 3 in CVL compared to controls [99]. Moreover, the defensin-mediated recruitment of innate and adaptive immune cells in response to preexisting genital infections may facilitate HIV-1 dissemination and replication by increasing the number of available target cells [100]. Although HBDs exhibit anti-HIV-1 activity, these studies suggest that a multitude of other factors in the female genital tract including differences in the vaginal microbiome and presence of additional sexually transmitted infections may alter the role of HBDs *in vivo*. Such discrepancies emphasize the need for further research on the complex interactions between genital infections, innate immunity, adaptive immunity, and HIV-1 pathogenesis.

The variable expression of HBDs in epithelial cells of different origins ultimately impacts how these peptides function to protect the host from infectious agents. While *β*-defensins exhibit strong anti-HIV-1 activity in oral epithelial cells, in the GI tract, their association with increased secretion of proinflammatory molecules may promote local inflammation and viral transmission. Future research efforts on the relationship between HBDs and HIV-1 should focus on (1) elucidating the mechanisms involved in reduced virion infectivity by *β*-defensins in the oral environment and assessing their potential to limit HIV-1 spread in other epithelial tissues and (2) exploring the human genomic sequence for novel HBDs. To date, over 50 *β*-defensins have been identified at the genomic and transcriptional level in humans, although only four HBDs (HBD1–4) have been well characterized at the protein and functional level [101–104]. The two *β*-defensin isoforms HBD5 and 6 were found to be exclusively expressed in the human epididymis [28], and *β*-defensins HBD25–29, also known as DEFB125–129, appear to be similarly predominantly expressed in the male genital tract [27]. Recombinantly produced HBD5 and HBD6 exhibited antimicrobial activity against *E. coli* [105], suggesting that these peptides may also promote host protection and innate immunity. Additional roles may be assigned to sperm maturation and transport impacting male fertility as it had been suggested for DEFB126 [106].

## 4. *θ*-Defensins

### 4.1. Immunomodulatory Properties

*θ*-Defensins are naturally expressed in Old World monkeys and are the only cyclic peptide identified in mammals [107]. Six isoforms, RTD1–6, have been isolated from neutrophils and bone marrow of nonhuman primates [108, 109]. Like most defensins, RTDs have potent antimicrobial activity, but, compared to *α*- and *β*-defensins, *θ*-defensin activities are relatively insensitive to salt, divalent cations, and serum [110–112]. Although human bone marrow expresses mRNA similar to the mRNA precursor of RTDs, defensin peptides are not synthesized naturally. Using solid-phase peptide synthesis, researchers have synthesized homologous RTD peptides referred to as retrocyclins according to the human pseudogene sequences [107]. In addition to their antimicrobial and possibly protease-inhibiting activities [113, 114], *θ*-defensins also exhibit immunomodulatory properties. Unlike *α*- and *β*-defensins, the *θ*-defensin RTD1 suppresses the secretion of proinflammatory cytokines by inhibiting the activation of NF-*κ*B and MAPK pathways [32], and RTD1 treatment in mice reduced the levels of proinflammatory cytokines in blood leukocytes that correlated with increased survival of bacterially infected animals [115]. RTD1-treated mice exposed to a mouse severe acute respiratory syndrome (SARS) coronavirus also showed increased survival associated with decreased levels of proinflammatory cytokines [116]. Interestingly, viral titers remained high throughout the study, suggesting that RTD1 increased survival without inactivating viral particles. RTD1 may also inhibit TNF*α*-converting enzyme [114] possibly adding to the immunosuppressive activity of RTD1. This protective activity of *θ*-defensins is unique compared to that of other defensins in which it stems from a pronounced suppression of proinflammatory responses, rather than direct interaction with the pathogens [115]. Because excess production of proinflammatory cytokines induced by HIV-1 exacerbates disease progression, the potential for *θ*-defensins to mediate these responses needs to be assessed. On the other hand, the expression of functional *θ*-defensins in Old World monkeys and their resistance to HIV-1 may be a causal relationship, and the anti-inflammatory characteristics of *θ*-defensins should be evaluated to determine if these peptides can reduce inflammation and subsequent epithelial impairment resulting from HIV-1 infection and can be used to combat HIV in humans.

### 4.2. Anti-HIV Properties

**Figure 1:**
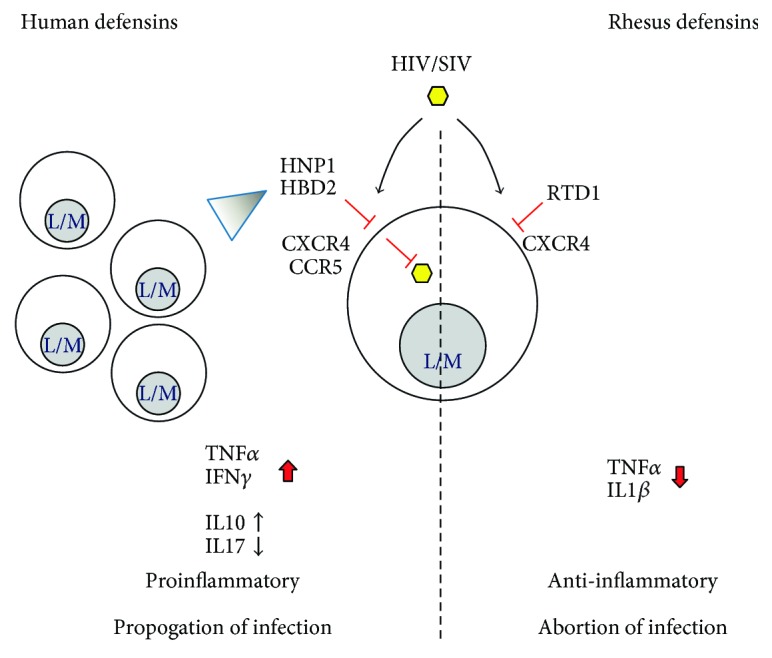
Model of the differential action of human and rhesus defensins with antiretroviral activity. The actions of the best-described human defensins with antiretroviral activity, human neutrophil peptide 1 (HNP1) and human beta-defensin 2 (HBD2) (left side) are contrasted to the actions of rhesus theta defensin 1 (RTD1) (right side) leading to the proposed differential immune response and outcome of the retrovirus infection. HIV and SIV represent human immunodeficiency or simian immunodeficiency virus, respectively, shown with yellow hexagons. CXCR4 and CCR5 are the chemokine coreceptors mediating virus entry on susceptible cells, in particular, lymphocytes, monocytes, and macrophages (L/M). Red bars: inhibitory activity at the level of virus entry or viral replication. Triangle: chemotactic activity. Defensin-mediated changes in the levels of key cytokines are shown with the proposed effects on the overall immune response and outcome of the viral infection.

Both natural and synthetic *θ*-defensins possess anti-HIV-1 activity [117–119]. Like HNP1 and HBD2, RTD1 downmodulates CXCR4 and inhibits the entry of CXCR4 tropic HIV-1. However, HNP1 and HBD2 also block viral replication after cDNA formation and inhibit CCR5 tropic HIV-1, indicating that distinct mechanisms are utilized by cyclic and acyclic defensins [30]. Key differences in antiretroviral and immune modulatory activities of selected human defensins and rhesus *θ*-defensins are highlighted in Figure 1. The synthetic RC1 reduces infectivity by preventing the formation of proviral DNA [119] and blocking HIV-1 Env-mediated cell fusion [120]. Furthermore, RC1 provides more protection to CD4^+^ T cells from both T cell tropic and M-tropic HIV-1 strains than do RTD1, 2, or 3 [119]. RCs can further inhibit viral entry by binding viral gp120 and CD4 receptors, where RC2 exhibits the strongest binding affinity compared to other RCs [41]. These studies suggest that RTDs and RCs reduce HIV-1 infectivity by disrupting viral entry instead of directly inactivating virions, as observed for HNPs and HBDs.

Recent research has focused on the use of RC congeners to develop anti-HIV-1 microbicides that prevent viral transmission. Analogues of RC1 have been engineered to enhance antiviral properties, with the analog RC101 demonstrating greater inhibitory activity against HIV-1 than RC1 [117]. As such, RC101 has been tested for use as a microbicide in vaginal tissues, and the results are promising. A quick-dissolving film formulated with RC101 showed anti-HIV-1 activity *in vitro*, was nontoxic in reproductive tissues, and remained bioactive for up to 6 months [121, 122]. Moreover, RC101 treatment inhibited pathogenic bacteria in vaginal tissues that are associated with bacterial vaginosis, a condition that increases HIV-1 susceptibility, while simultaneously maintaining beneficial microbial communities that protect the host from infection [123]. The lack of toxicity *in vivo*, the compatibility of RC101 with normal vaginal microbiota, and the ability of RC101 to inhibit pathogenic bacterial growth make RC101 an ideal peptide for anti-HIV-1 microbicide development. Research and development of inexpensive anti-HIV-1 microbicides are especially important in countries with high infection rates and limited resources. Topical microbicides that prevent HIV-1 transmission could dramatically reduce the number of new infections and deaths resulting from AIDS each year. Future investigations should continue to assess the use of RC101 as a safe, effective microbicide, with an eventual transition into clinical trials.

## 5. Conclusion

**Figure 2:**
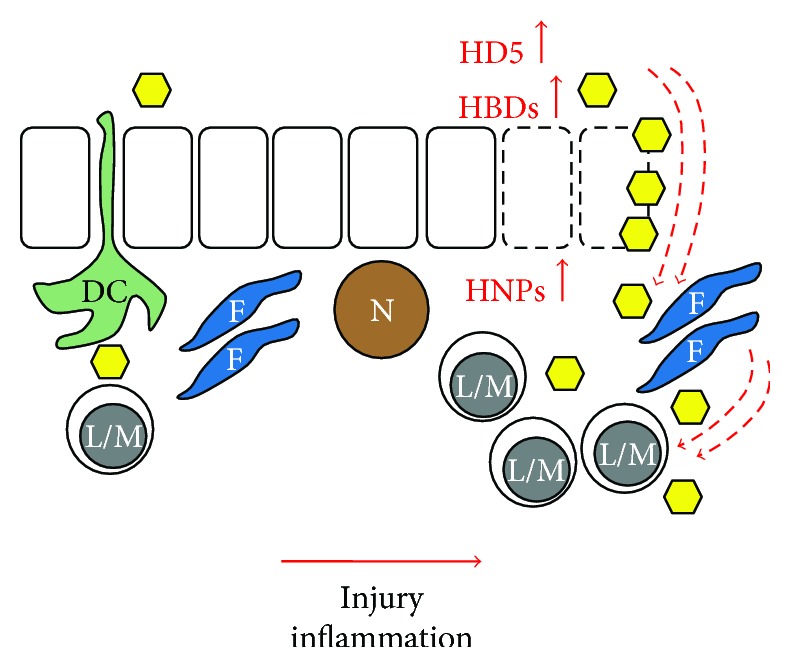
Impact of defensins on HIV infection. Dendritic cells (DC) sampling mucosal surfaces deliver HIV (yellow hexagon) to mononuclear cells (L/M, lymphocytes and monocytes/macrophages). Epithelial cell injury with subsequent inflammatory responses or inflammation of other causes (i) activates epithelial cells (rectangles with solid lines) to increase the production of human defensin 5 (HD5) and *β*-defensins (HBDs) and (ii) recruits neutrophils (N) which deliver human neutrophil peptides (HNPs). HNPs impair the epithelial cell barrier (rectangles with dotted lines) and HD5, supported by HBDs, and enhance HIV influx which is amplified by stromal fibroblasts (F). Simultaneously, the chemotactic properties of HNP1 and HBD2 have effected an influx of susceptible mononuclear cells further aggravating the HIV infection.

Defensins comprise a critical component of the innate immune response, exhibit activities against a broad range of pathogens, shape the normal microbiota, and modulate immune responses. As HIV-1 preferentially targets cells associated with adaptive immunity, the innate immune response is critical and research continues to explore the ability of defensins to limit viral transmission and to mediate the effects of disease progression. The anti-HIV-1 activity of defensins works on several levels: direct inactivation of virions, inhibition of viral entry, interference of viral replication following cell entry, increasing the production of other antiviral factors, and facilitation of cellular communications that regulate adaptive responses. On the other hand, the immunomodulatory action of defensins may also impact the epithelial barrier function, thereby contributing to HIV dissemination through increased uptake of HIV via stromal fibroblasts and recruitment of susceptible target cells (Figure 2). The SIV-infected macaque model is a well-accepted model for the study of HIV pathogenesis. Continuing to employ the macaque model of SIV infection, combined with insights gained from studies in other diseases on defensin synthesis and immunomodulatory characteristics, is an important key to future progress in HIV research. One crucial next step includes further research into the development of defensin-based HIV-1 therapeutics. The successful use of RC101 in topical microbicides to reduce HIV-1 transmission is encouraging, and research efforts must continue to explore effective, inexpensive ways to decrease the rate of new infections. Targeting the immunomodulatory action of defensins on the GI barrier opens new approaches for limiting HIV dissemination and disease progression in infected patients.

## Figures and Tables

**Table 1:** Cellular sources and tissue localization of *α*-defensins.

	HNP1–3	HNP4^a^	HD5	HD6^a^	References
Cellular (primary)	Neutrophils	Neutrophils	Paneth cells	Paneth cells	[23, 24, 42, 47, 124–128]
Cellular (also reported)	Monocytes, NK cells, B cells, *γδ* T cells, intestinal epithelial cells		Female reproductive tract epithelial cells, urinary tract epithelial cells		
Tissue^b^	Cervical mucus plug, spleen, thymus				

^a^Limited data available due to lack of availability of antibodies. ^b^mRNA expression was identified in tissue but cellular source was not determined.

**Table 2:** Cellular sources and tissue localization of selected *β*-defensins.

	HBD1	HBD2	HBD3	HBD4	HBD5/6	References
Cellular (primary)	Keratinocytes, kidney epithelial cells, airway epithelial cells, female reproductive tract epithelial cells, mammary epithelial cells	Keratinocytes, oral epithelial cells	keratinocytes, airway epithelial cells, oral epithelial cells	Keratinocytes, airway epithelial cells		[28, 75, 128–134]
Cellular (also reported)	Monocytes, macrophages, dendritic cells	Monocytes, macrophages, dendritic cells		Neutrophils		
Tissue^a^	Pancreas	Trachea, lung	Tonsil, skin	Lung, kidney, uterus, testis, gastric antrum	Epididymis	

^a^mRNA expression was identified in tissues but cellular source was not determined.

**Table 3:** Cellular sources of *θ*-defensins.

RTD1	RTD2-3	RTD4–6	References
Monocytes, neutrophils, myeloblasts, Paneth cells	Monocytes, neutrophils, myeloblasts	Neutrophils, myeloblasts	[29, 31, 109, 135, 136]
